# Comparison of regional soft tissue changes after bimaxillary rotational surgery between class III deformity with overbite and open bite: A 3D imaging analysis

**DOI:** 10.1016/j.bj.2022.09.003

**Published:** 2022-09-29

**Authors:** Piyanan Keardkhong, Yun-Fang Chen, Chuan-Fong Yao, Ying-An Chen, Yu-Fang Liao, Yu-Ray Chen

**Affiliations:** a Graduate Institute of Dental and Craniofacial Science, College of Medicine, Chang Gung University, Taoyuan, Taiwan; b Craniofacial Center, Chang Gung Memorial Hospital, Taoyuan, Taiwan; c Craniofacial Research Center, Chang Gung Memorial Hospital at Linkou, Taoyuan, Taiwan; d Department of Craniofacial Orthodontics, Chang Gung Memorial Hospital at Taipei, Taipei, Taiwan; e Department of Plastic and Reconstructive Surgery, Chang Gung Memorial Hospital at Linkou, Taoyuan, Taiwan; f Department of Craniofacial Orthodontics, Chang Gung Memorial Hospital, Taoyuan, Taiwan

**Keywords:** 3D, Soft tissue, Class III malocclusion, Orthognathic surgery

## Abstract

**Background:**

This prospective study aimed to compare regional soft tissue changes between patients with class III overbite and open bite deformities treated with bimaxillary surgery involving clockwise and counter-clockwise mandibular setback, respectively.

**Material and methods:**

Class III deformity adults receiving Le Fort I and bilateral sagittal split osteotomies were grouped according to the incisal occlusion: overbite (n = 30) and open bite (n = 30). Combined cone-beam CT scans and 3D facial photographs preoperative and at least 1-year postoperative were taken to assess the soft tissue changes.

**Results:**

Postoperative changes for the overbite and open bite groups included anterior repositioning of nose (−0.8 ± 1.2 mm and −1.1 ± 1.1 mm, respectively) and cheek (−1.9 ± 1.3 mm and −1.7 ± 2.6 mm, respectively), posterior repositioning of chin (5.2 ± 4.0 mm and 4.9 ± 3.2 mm, respectively), and medial (−1.7 ± 2.0 mm and −1.9 ± 2.1 mm, respectively) and posterior (2.7 ± 1.4 mm and 2.8 ± 2.3 mm, respectively) repositioning of bilateral angles. Posterior (1.2 ± 2.0 mm and 5.1 ± 3.3 mm) and inferior (−1.4 ± 2.2 mm and −2.4 ± 2.7 mm) repositioning of upper lip and lower lip occurred in overbite group. Inferior (−2.3 ± 2.4 mm) and superior (3.7 ± 3.4 mm) repositioning of chin occurred in the overbite and open bite groups, respectively.

**Conclusions:**

Treatment of class III overbite and open bite deformities with bimaxillary rotational surgery resulted in comparable regional soft tissue changes, except for upper lip, lower lip and chin.

**At a glance commentary**

**Scientific background on the subject**

This study evaluated patients with class III deformity and mandibular prognathism. Bimaxillary rotational surgery using either clockwise or counter-clockwise mandibular setback was performed for patients with overbite or open bite respectively.

**What this study adds to the field**

The manipulation of the maxillomandibular complex in orthognathic surgery has been used in the treatment of class III deformity in order to optimize aesthetic and functional results. Postoperative soft tissue changes were comparable except for the upper lip, lower lip and chin between patients with overbite and open bite. These findings provide clinicians with a better understanding of the orthognathic outcomes on different facial regions for patients with class III deformity.

Skeletal class III deformity is characterized by cheek depression and mandibular protrusion and is a frequently encountered skeletal deformity for Asian populations [1]. The severe jaw discrepancy can be effectively corrected with bimaxillary orthognathic surgery (OGS), which alters the maxillomandibular deformity associated with the dental malocclusion through osteotomy and repositioning the maxilla and mandible to achieve an ideal occlusion and facial appearance. Bimaxillary rotational surgery with mandibular setback is the preferred therapy for correction of skeletal class III deformity for patients in Asian populations because it provides a better aesthetic outcome with increased stability [[2], [3], [4], [5]]. Although the upper airway volume decreases after the rotational surgery, it is not smaller than in normal controls because the initial upper airway in patients with class III deformity is enlarged due to an oversized mandible compared with that of normal controls [6]. The technique employs Le Fort I posterior impaction osteotomy followed by autorotation and setback of bilateral sagittal split osteotomy (BSSO); clockwise rotation for overbite cases and counter-clockwise rotation for open bite cases; genioplasty is also performed if necessary. Patients are more concerned with improving their facial features than with the underlying skeletal changes, thus, evaluating the soft tissue response following the maxillomandibular movement is crucial [7,8].

Soft tissue changes after bimaxillary OGS for class III deformity have been assessed in two- and three-dimensions (2D and 3D, respectively). The 2D methods use superimposed preoperative and postoperative images from lateral cephalometry to measure changes in selected landmarks on both hard and soft tissues. Consequently, the focus of the soft tissue response has been on the linear and angular measurements of certain facial landmarks in the mid–sagittal plane [[9], [10], [11], [12]]. Cone beam computed tomography (CBCT) [[13], [14], [15], [16], [17], [18], [19]], laser scanning [[20], [21], [22]], and stereophotogrammetry [3,[23], [24], [25]] have allowed further assessment of postsurgical changes following bimaxillary surgery for class III deformity using 3D images of hard or soft tissues of facial structures.

Unfortunately, most 3D studies have examined changes following maxillary advancement and mandibular setback [17,[21], [22], [23], [24], [25]], which are different from bimaxillary rotational surgery. In addition, these studies used only 3D landmarks to evaluate the 3D regional soft tissue changes on the nose, cheek, upper and lower lips, or chin [17,[21], [22], [23]]. These 3D landmarks do not comprehensively address the physical 3D curvature of facial changes [27,28]. Although the use of CBCT data for orientating and superimposing serial head models shows high reproducibility, the CBCT-rendered soft tissue model has been reported to be of lower quality than that of 3D photographs [29,30].

Therefore, the purpose of this study was to compare regional 3D changes in facial soft tissues after bimaxillary rotational surgery for patients receiving either clockwise or counter-clockwise mandibular setback for correction of skeletal class III overbite and open bite deformities, respectively. Changes were assessed using a combination of CBCT and 3D stereophotogrammetric images obtained presurgery and at least 1-year postsurgery. The null hypothesis of this study was that there was no difference in any of the soft tissue measurements between groups.

## Materials and methods

### Patients

The protocol for this prospective study was approved by the hospital's Institutional Ethics Committee. Sixty adult patients (age ≥18 years) with skeletal class III deformity (A point-nasion-B point angle ≤0°) and no significant facial asymmetry (menton deviation <4 mm and no significant contour asymmetry) who underwent at least a Le Fort I osteotomy and a BSSO using a surgery-first approach by the same team of attending surgeons, and post-surgical orthodontic treatment by a single orthodontist at the Chang Gung Craniofacial Center during a 3-year period were consecutively recruited. Exclusion criteria were patients who had a history of craniofacial surgery, a craniofacial anomaly, cleft lip or cleft palate, or had undergone multi-segmental Le Fort I osteotomy, jaw contouring or rhinoplasty during the bimaxillary surgery or before orthodontic debonding.

Patients’ age, sex, body mass index, and additional genioplasty were recorded. The 60 patients were divided into two groups according to the incisal occlusion. Overbite group consisted of patients showing positive overbite whose mandibular occlusal plane (MOP) had surgical clockwise rotation immediately after surgery (value of SN-MOP increased), and open bite group was open bite patients undergoing counter-clockwise rotation (value of SN-MOP decreased).

### Surgical procedure

The surgical procedure consisted of Le Fort I posterior impaction and BSSO setback with or without genioplasty. We performed maxilla-first surgical sequence for the 2-jaw surgery. After Le Fort I osteotomy, maxillomandibular fixation onto the intermediate splint guided the new maxillary position. As premature contact came from the posterior maxilla, special care was taken to remove all the bony interferences located between the pterygoid plates and tuberosities of the maxilla [31]. Rigid fixation of maxilla over 4 buttresses was applied after the planned vertical position of maxilla achieved. The BSSO was performed using the short-split technique [32,33]. To allow easier setback of the mandibular distal segment, the pterygo-masseteric sling was detached completely. After securing the position of the distal segment with intermaxillary fixation, premature contact between the proximal and distal segments was removed using an oscillating saw or burr until satisfactory bone contact between the two segments was achieved. Mono-cortical titanium bone plates and screws were used for rigid fixation. Genioplasty was performed as needed based on intra-operative assessment of soft tissue profile and proportion.

### Post-surgical orthodontic treatment

The goals of post-surgical orthodontic treatment in surgery-first orthognathic approach are to decompensate the malocclusion, detail the occlusion, and ensure the skeletal stability [2]. One month before surgery, 0.022-in. preadjusted brackets were bonded. To ensure that there was no problem in fitting the splints, 0.014-in. or 0.016-in. nickel-titanium archwires were not inserted until 1–3 days before surgery.

Alignment, leveling and coordination were initiated immediately after surgery because the active archwires (i.e., nickel-titanium archwires) were already left in place in order to take advantage of the unlocked occlusion and rapid tooth movement following surgery. Once alignment, leveling, and coordination were almost achieved, 0.016 × 0.022-in. nickel-titanium archwires were replaced with 0.016 × 0.022-in. stainless steel archwires. Incomplete incisor decompensation could be assisted by class II elastics; incomplete arch coordination could be assisted by a transpalatal arch or elastics. By this time the post-surgical orthodontic finishing was much the same as with post-surgical orthodontic treatment in classic orthognathic approach (i.e., orthodontics-first).

### CBCT

CBCT of the head and neck was performed presurgery (T0) and postsurgery (T1, at orthodontic debonding and at least one year after surgery) using an i-CAT 3D Dental Imaging System (Imaging Sciences International, Hatfield, PA, USA) with the following parameters: 120 kVp, 0.4 × 0.4 × 0.4-mm voxel size, 40-s scan time, and 20 × 20-cm field of view. The patient's head was positioned with the Frankfort horizontal (FH) plane parallel to the ground. Throughout the scan, the patient was asked not to swallow. The patient was also instructed to keep their mouth closed and to maintain a centric occlusion bite.

**Fig. 1:**
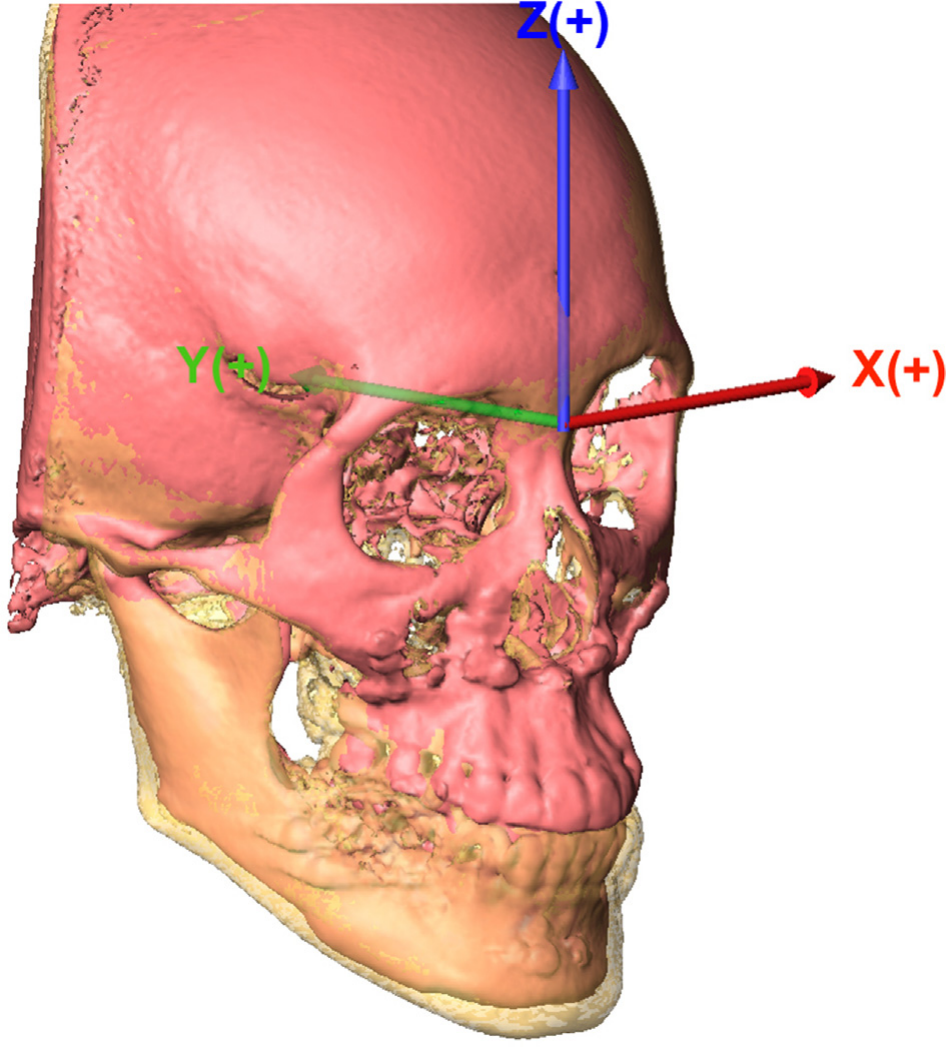
Representative superimposition of CBCT images showing changes in facial skeleton preoperative and postoperative (orange and yellow, respectively) using voxel-based registration of cranial structures and the 3D coordinates. X-axis (left/right): + = more to the left, − = more to the right; Y-axis (posterior/anterior): + = more posterior, − = more anterior; Z-axis (superior/inferior): + = more superior, − = more inferior.

Images were stored in Digital Imaging and Communications in Medicine format (DICOM) and rendered into volumetric images using AVIZO version 7.0 software (FEI, Mérignac, France). Sagittal, axial, and coronal slices and 3D reconstructions of images were used for analysis. All images were analyzed by a single investigator (PK) blinded to patients' treatment histories. Before analysis, 3D images were reoriented as follows: (1) the axial plane was the FH plane, defined by the best fit plane through the bilateral orbitale and porion; (2) the midsagittal plane was perpendicular to the FH plane, passing through the nasion and basion; and (3) the coronal plane was perpendicular to the FH and coronal planes, passing through the nasion. Patients’ cranial structures not affected by surgery were used to superimpose preoperative and postoperative CBCT images using voxel-based registration to position images in the same 3D coordinates [Fig. 1].

After registration of the 3D images, ten hard tissue landmarks were located by the same investigator: sella (S), nasion (N), anterior nasal spine (ANS), A point, posterior nasal spine (PNS), B point, upper central incisor (U1), lower central incisor (L1) and lower first molar (L6).

### 3dMD images

Digital 3D photographs of the head were captured by using the 3dMDface System, which generates a 3D image of the area from at least two planes (3dMD Inc., Atlanta, GA, USA). Preoperative (T0) and postoperative (T1) images were obtained on the same day as the CBCT images. During the scan, patients were placed with the head in the same natural position as for the CBCT scans and the patient was also instructed to keep eyes opened, mouth closed, and bite in a centric occlusion.

Models of the 3D photographs of face (excluding artifacts such as the eyes, nose, mouth) were superimposed on the 3D CBCT models with the same 3D coordinates using surface-based registration. The mean error by calculating the root-mean-square deviation (RMSD) in superimposition was 0.39 ± 0.06 mm (range: 0.28 to 0.50), indicating superimposition had a high level of accuracy. Superimposed preoperative (T0) and postoperative (T1) images were compared to determine changes in soft tissue structures.

**Fig. 2:**
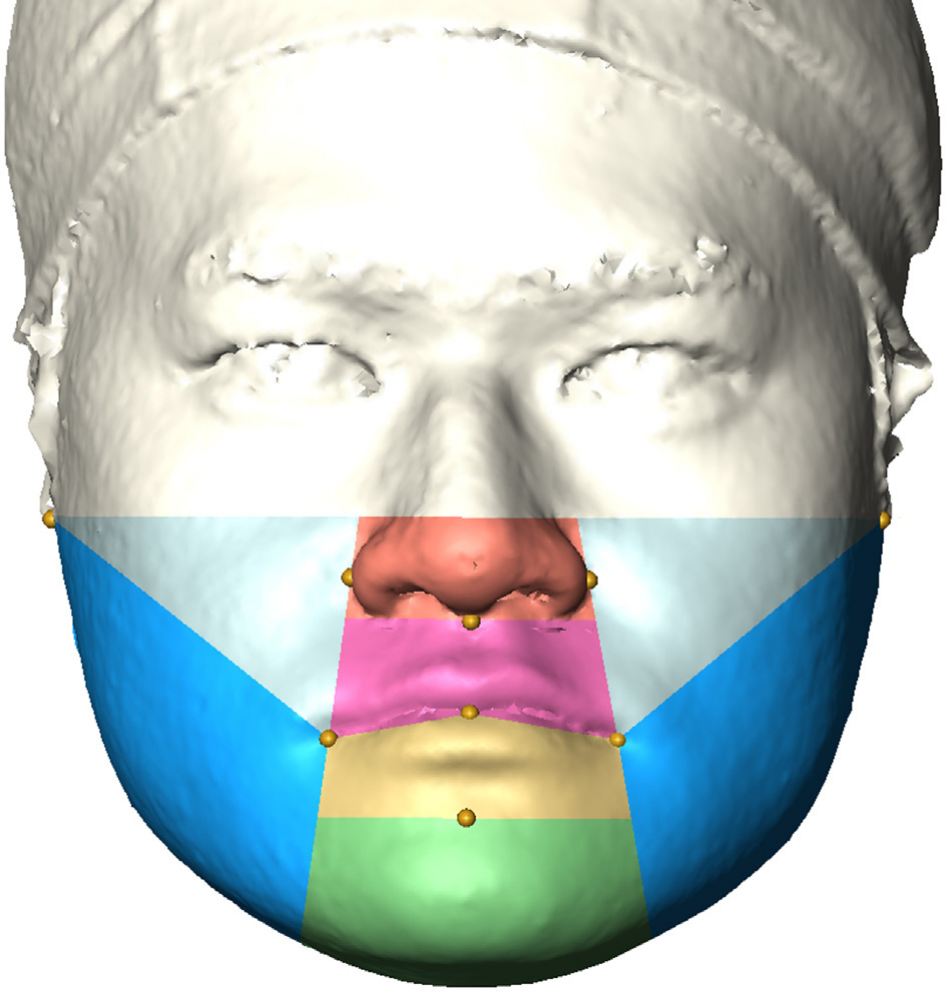
3D model following surface-based registration showing the eight facial regions used in this study: nose (orange), right and left cheek (light blue), upper lip (pink), lower lip (yellow), angle (blue), and chin (green).

After surface registration of the 3D images, 12 soft tissue landmarks (Rt.Tra, Lt.Tra, Rt.Al, Lt.Al, Sn, Stm, Rt.Ch, Lt.Ch, B′ point, Me’, Rt.Go’, Lt.Go’) and 11 reference planes were located on the 3D surface model to divide the face into eight regions (nose, right cheek, left cheek, upper lip, lower lip, chin, right angle, left angle) (Table 1 and Fig. 2) [34].

**Table 1:** Soft tissue landmarks and reference planes used for definition of face regions.

Soft tissue	Definition
Landmark	
Rt.Tra (right tragus)	The point at the upper margin of the right tragus
Lt.Tra (left tragus)	The point at the upper margin of the left tragus
Rt.Al (right alar base)	The most lateral point the curved base line of alar on the right side
Lt.Al (left alar base)	The most lateral point the curved base line of alar on the left side
Sn (subnasale)	The midpoint on the nasolabial soft tissue contour between the columella crest and the upper lip
Stm (stomion)	The point at the midline of labial fissure between gently closed lips
Rt.Ch (right cheilion)	The point located at right labial commissure
Lt.Ch (left cheilion)	The point located at left labial commissure
B’ (soft tissue B point)	The most posterior midpoint on the labiomental soft tissue contour
Me’ (soft tissue menton)	The most inferior midpoint of the chin
Rt.Go’ (right soft tissue gonion)	The most lateral point on the soft tissue contour of right mandibular angle
Lt.Go’ (left soft tissue gonion)	The most lateral point on the soft tissue contour of left mandibular angle
Reference plane	
Upper nasal plane	A plane through landmarks Rt.Tra and Lt.Tra and perpendicular to the coronal plane
Lower nasal plane	A plane through landmark Sn and parallel to the upper nasal plane
Lateral right nasal plane	A plane through landmarks Rt.Al and Rt.Ch and perpendicular to the coronal plane
Lateral left nasal plane	A plane through landmarks Lt.Al and Lt.Ch and perpendicular to the coronal plane
Right lip plane	A plane through landmarks Rt.Ch and Stm and perpendicular to the coronal plane
Left lip plate	A plane through landmarks Stm and Lt.Ch and perpendicular to the coronal plane
Right cheek plane	A plane through landmarks Rt.Tra and Rt.Ch and perpendicular to the coronal plane
Left cheek plane	A plane through landmarks Lt.Tra and Lt.Ch and perpendicular to the coronal plane
Upper chin plane	A plane through landmark B′ and parallel to the upper nasal plane
Lateral right chin plane	A plane through landmarks Rt.Al and Rt.Ch and perpendicular to the coronal plane
Lateral left chin plane	A plane through landmarks Lt.Al and Lt.Ch and perpendicular to the coronal plane

### Hard tissue changes

Changes in hard tissue (maxilla, mandible, and dentition) after surgery were assessed by the preoperative (T0) to postoperative (T1) changes in nine variables. These variables included maxillary protrusion (SNA), mandibular protrusion (SNB), jaw relation (ANB), palatal plane angle (SN-PP, angle formed by sella-nasion line and palatal plane), mandibular plane angle (SN-MP, angle formed by sella-nasion line and mandibular plane), MOP angle (angle formed by sella-nasion line and MOP), upper incisal angle (SN-U1), lower incisal angle (L1-MP), overjet and overbite.

### Soft tissue changes

**Fig. 3:**
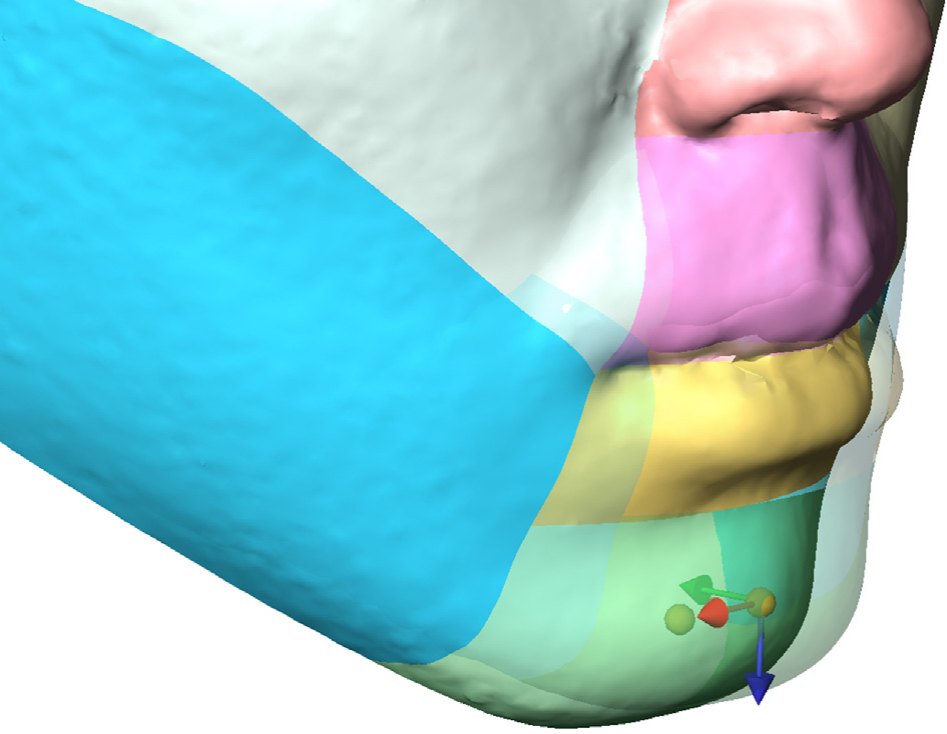
Representative image of a patient of overbite group illustrating the distance and direction of change in the chin centroid. Red indicates the X-axis, green indicates the Y-axis, and blue indicates the Z-axis. For this image, movement in the X-axis was 2 mm to the right; movement in the Y-axis was 9.4 mm posterior; and movement in the Z-axis was 3 mm inferior.

Changes in soft tissue width (nose, mouth and bi-gonion) after surgery were assessed by the preoperative (T0) to postoperative (T1) linear changes. Changes in eight facial regions (nose, right cheek, left cheek, upper lip, lower lip, chin, right angle, left angle) were assessed by the preoperative (T0) to postoperative (T1) changes in the surface area, distance and direction of the centroids in three coordinates: X-axis (left/right): + = more to the left, − = more to the right; Y-axis (posterior/anterior): + = more posterior, − = more anterior; and Z-axis (superior/inferior): + = more superior, − = more inferior [Fig. 3]. The centroids (coordinates of the center of the surface object) were determined via automatic computation using AVIZO program after the eight facial regions were defined.

### Reliability

To assess intra-examiner error, all the combined CBCT/3dMD measurements were repeated by the same investigator for 10 randomly chosen patients in clockwise group (n = 5) and counter-clockwise group (n = 5); measurements were separated by two weeks. The intra-class correlation coefficient (ICC) was used to determine reliability. The mean ICC value was 0.952 (95% confidence interval, 0.903 to 0.981) indicating excellent intra-examiner reliability.

### Statistical analysis

Statistical analysis was performed with the statistical software package SPSS version 23.0 for Windows (SPSS INC, Chicago, USA). The Kolmogorov–Smirnov test was implemented to verify the normality of the distribution of the data. Descriptive statistics were expressed as means ± standard deviation (SD) for metric variables and as frequency and percentage for nominal variables. Patient's clinical characteristics and CBCT/3dMD preoperative and postoperative measurements were compared using paired t-test or Wilcoxon signed rank test for data with or without normal distribution, respectively. Independent t-test or Mann–Whitney U test compared the differences between the two groups for data with or without normal distribution, respectively. Probabilities of less than 0.01 were accepted as significant in order to account for multiple comparisons.

## Results

### Patients

In this study, 30 overbite patients underwent clockwise mandibular setback (overbite group: mean age, 23.7 ± 5.4 years; mean surgical rotation of MOP, 5.1 ± 4.7°, data not shown) and 30 open bite patients underwent counter-clockwise mandibular setback (open bite group: mean age, 22.6 ± 4.6 years; mean surgical rotation of MOP, −3.4 ± 3.1°, data not shown). The patient's characteristics before treatment showed no significant differences except overbite and SN-MP angle. All patients in overbite group had positive overbite (2.5 ± 2.3 mm), while open bite group was negative overbite (open bite, −1.8 ± 2.6 mm). The baseline characteristics of the patients before surgery are listed in Table 2.

**Table 2:** Demographics and preoperative clinical characteristics of overbite (n = 30) and open bite (n = 30) patients receiving clockwise and counter-clockwise rotational surgery, and differences between the two groups.

	Overbite group		Open bite group		Difference
Characteristic	n (%)	Mean (SD)	n (%)	Mean (SD)	*p*-value
Female	16 (53)		16 (53)		0.999
Age at surgery, years		23.7 (5.4)		22.6 (4.6)	0.400
Postoperative follow-up, months		18.0 (4.9)		17.6 (6.8)	0.760
Genioplasty	21 (70)		22 (73)		0.999
Body mass index, kg/m^2^		21.2 (3.2)		21.7 (2.9)	0.531
SNA, degrees		80.8 (1.6)		79.9 (2.1)	0.054
SNB, degrees		85.1 (3.4)		83.9 (3.1)	0.264
ANB, degrees		−4.2 (3.2)		−4.0 (3.2)	0.820
SN-PP, degrees		12.5 (4.8)		14.3 (5.7)	0.188
SN-MOP, degrees		11.6 (5.1)		22.1 (5.0)	<0.001∗∗∗
SN-MP, degrees		33.3 (8.0)		39.4 (4.7)	0.001∗∗
SN-U1, degrees		110.1 (13.6)		107.0 (6.0)	0.265
L1-MP, degrees		77.9 (9.7)		77.8 (11.2)	0.959
Overjet, mm		−3.2 (3.4)		−3.1 (3.5)	0.877
Overbite, mm		2.5 (2.3)		−1.8 (2.6)	<0.001∗∗∗

Abbreviations: SD: standard deviation; SNA: sella-nasion-A point; SNB: sella-nasion-B point; ANB: A point-nasion-B point; SN: sella-nasion; PP: palatal plane; MOP: mandibular occlusal plane; MP: mandibular plane; U1: upper central incisor; L1: lower central incisor.

∗∗*p* < .01, ∗∗∗*p* < .001; significance determined by chi-square test, Mann–Whitney U test or independent t-test.

### Hard tissue changes

After surgery, the SNB and upper incisal angle (SN-U1) decreased while the ANB, SN-PP, lower incisal angle (L1-MP) and overjet increased in both groups. The SNA decreased in overbite group; the overbite increased in open bite group. The increase of SN-MP was more in overbite group than in open bite group [Table 3].

**Table 3:** Postoperative changes in facial hard tissue variables: mean difference between T1 and T0 for overbite (n = 30) and open bite (n = 30) groups, and difference between groups.

Variable^a^	Overbite group				Open bite group				Difference (T1–T0)
	T0	T1	T1–T0	*p*-value	T0	T1	T1–T0	*p*-value	*p*-value
	Mean (SD)	Mean (SD)	Mean (SD)		Mean (SD)	Mean (SD)	Mean (SD)		
SNA, degrees	80.8 (1.6)	79.6 (1.6)	−1.2 (2.0)	0.003∗∗	79.9 (2.1)	79.2 (1.5)	−0.7 (2.5)	0.011	0.425
SNB, degrees	85.1 (3.4)	77.7 (1.1)	−7.4 (3.1)	<0.001∗∗∗	83.9 (3.1)	77.6 (1.6)	−6.4 (3.0)	<0.001∗∗∗	0.224
ANB, degrees	−4.2 (3.2)	2.0 (2.1)	6.2 (2.5)	<0.001∗∗∗	−4.0 (3.2)	1.6 (2.5)	5.7 (3.4)	<0.001∗∗∗	0.505
SN-PP, degrees	12.5 (4.8)	16.2 (4.1)	3.8 (4.5)	<0.001∗∗∗	14.3 (5.7)	18.6 (6.1)	4.3 (7.7)	0.001∗∗	0.842
SN-MOP, degrees	11.6 (5.1)	16.9 (5.7)	5.3 (5.1)	<0.001∗∗∗	22.1 (5.0)	18.0 (3.8)	−4.2 (3.6)	0.001∗∗	<0.001∗∗∗
SN-MP, degrees	33.3 (8.0)	39.9 (6.5)	6.6 (6.3)	<0.001∗∗∗	39.4 (4.7)	41.4 (5.8)	2.0 (6.3)	0.033	0.006∗∗
SN-U1, degrees	110.1 (13.6)	107.9 (16.9)	−2.2 (8.2)	0.007∗∗	107.0 (6.0)	104.4 (8.5)	−2.6 (7.9)	0.002∗∗	0.057
L1-MP, degrees	77.9 (9.7)	82.4 (7.9)	4.5 (7.6)	0.003∗∗	77.8 (11.2)	82.1 (7.4)	4.4 (9.0)	0.012	0.947
Overjet, mm	−3.2 (3.4)	2.3 (0.6)	5.5 (3.3)	<0.001∗∗∗	−3.1 (3.5)	2.6 (0.8)	5.6 (3.6)	<0.001∗∗∗	0.636
Overbite, mm	2.5 (2.3)	1.6 (0.6)	−0.9 (2.4)	0.070	−1.8 (2.6)	1.9 (0.7)	3.6 (2.3)	<0.001∗∗∗	<0.001∗∗∗

Abbreviations: SD: standard deviation; T0: preoperative; T1: at least 1-year postoperative.

∗∗*p* < .01, ∗∗∗*p* < .001; significance between T0 and T1 and between groups determined by Mann–Whitney U test or independent t-test.

a Positive value indicates increase in measurement; negative value indicates decrease in measurement.

### Linear and surface area changes in soft tissues

There was a significant decrease after surgery in the bi-gonial width in both overbite and open bite groups (*p* < .001). In both groups, there were significant increases in surface areas of the cheek (*p* < .001) and decreases in surface areas of the bilateral angles and chin (*p* < .01 to *p* < .001). There was no significant difference between groups for any of the variables [Table 4].

**Table 4:** Postoperative changes in width and surface areas of soft tissues: mean difference between T1 and T0 for overbite (n = 30) and open bite (n = 30) groups, and difference between groups.

Variable	Overbite group				Open bite group				Difference (T1–T0)
	T0	T1	T1–T0	*p*-value	T0	T1	T1–T0	*p*-value	*p*-value
	Mean (SD)	Mean (SD)	Mean (SD)		Mean (SD)	Mean (SD)	Mean (SD)		
Width^a^, mm									
Nose (Rt.Al-Lt.Al)	39.5 (3.4)	39.7 (3.0)	0.2 (1.4)	0.524	38.7 (2.9)	39.1 (2.8)	0.4 (1.8)	0.249	0.301
Mouth (Rt.Ch-Lt.Ch)	48.4 (3.6)	47.5 (3.4)	−0.8 (2.6)	0.092	47.5 (3.9)	47.3 (3.8)	−0.2 (4.3)	0.833	0.479
Bi-gonion (Rt.Go’-Lt.Go’)	119.6 (8.5)	117.1 (8.0)	−2.3 (2.9)	<0.001∗∗∗	119.0 (6.5)	115.9 (5.8)	−3.1 (4.1)	<0.001∗∗∗	0.429
Surface area^a^, mm^2^									
Nose	1701.7 (377.4)	1691.1 (350.1)	−10.6 (150.3)	0.701	1627.5 (363.9)	1622.6 (395.2)	−4.8 (144.4)	0.856	0.813
Cheek									
Right	1665.2 (334.7)	1727.3 (342.1)	62.2 (81.2)	<0.001∗∗∗	1624.9 (301.2)	1706.1 (322.6)	81.2 (105.2)	<0.001∗∗∗	0.074
Left	1731.7 (350.0)	1823.3 (335.6)	91.6 (83.6)	<0.001∗∗∗	1677.3 (276.6)	1772.0 (278.2)	94.8 (115.5)	<0.001∗∗∗	0.619
Total (right + left)	1698.4 (326.9)	1775.3 (322.6)	76.9 (65.8)	<0.001∗∗∗	1651.1 (274.7)	1739.1 (286.9)	88.0 (101.6)	<0.001∗∗∗	0.871
Upper lip	1232.5 (170.6)	1285.2 (158.2)	52.7 (114.3)	0.017	1234.2 (181.2)	1273.3 (195.1)	39.1 (95.7)	0.069	0.619
Lower lip	1043.9 (199.0)	978.8 (180.8)	−65.1 (132.8)	0.012	1148.2 (230.7)	1105.5 (182.6)	−42.7 (121.8)	0.065	0.498
Angle									
Right	7549.3 (901.4)	7162.4 (948.3)	−386.9 (418.2)	<0.001∗∗∗	7736.0 (771.8)	7418.7 (781.3)	−317.3 (327.7)	<0.001∗∗∗	0.476
Left	7540.8 (845.8)	7277.7 (932.9)	−263.1 (395.3)	0.001∗∗	7818.7 (646.7)	7379.9 (646.5)	−438.9 (477.8)	0.001∗∗	0.033
Total (right + left)	7545.0 (844.3)	7220.0 (915.0)	−325.0 (362.8)	<0.001∗∗∗	7777.4 (652.9)	7399.3 (665.9)	−378.1 (353.2)	<0.001∗∗∗	0.723
Chin	4055.6 (544.2)	3802.25 (526.0)	−253.4 (348.6)	0.001∗∗	3996.0 (783.9)	3720.4 (670.1)	−275.6 (446.6)	0.002∗∗	0.647

Abbreviations: SD: standard deviation; T0: preoperative; T1: at least 1-year postoperative.

∗∗*p* < .01, ∗∗∗*p* < .001; significance between T0 and T1 determined by Wilcoxon signed ranks test or paired t-test, and between groups determined by Mann–Whitney U test or independent t-test.

a Positive value indicates increase in measurement; negative value indicates decrease in measurement.

### Changes in position of soft tissues

Postoperative changes in the position of soft tissues are shown in Table 5. In both overbite and open bite groups, soft tissues were significantly repositioned after surgery: the right angle (*p* < .01) shifted left; the left angle shifted right (*p* < .01); and the total angle moved medially (*p* < .001); differences between groups were not significant. Posterior or anterior changes were significant for all soft tissues, with anterior repositioning of the nose and cheek and posterior repositioning of the chin and bilateral angles in both groups. There was a difference between groups for the upper lip: repositioning was 1.2 mm posterior (SD = 2.0) in overbite group and 1.1 mm anterior (SD = 1.7) for open bite group (*p* < .001). For both groups, there was significant superior repositioning of the angles (*p* < .001) and there were no differences between groups. There was inferior repositioning of the upper and lower lip in overbite group (*p* < .01 and *p* < .001, respectively). Repositioning of the lower lip was significantly different between groups: repositioning was 2.4 mm inferior (SD = 2.7) for overbite group (*p* < .001) compared with 0.8 mm superior (SD = 4.6) for open bite group. There was inferior (2.3 mm, SD = 2.4) and superior (3.7 mm, SD = 3.4) repositioning of the chin for overbite and open bite groups, respectively, and the difference between groups was significant (*p* < .001).

**Table 5:** Postoperative changes in position of facial soft tissues: mean difference between T1 and T0 for overbite (n = 30) and open bite (n = 30) groups, and difference between groups.

Position	Overbite group				Open bite group				Difference (T1–T0)
	T0	T1	T1–T0	*p*-value	T0	T1	T1–T0	*p*-value	*p*-value
	Mean (SD)	Mean (SD)	Mean (SD)		Mean (SD)	Mean (SD)	Mean (SD)		
Left/right, mm^a^									
Nose	0.6 (1.2)	0.8 (1.3)	0.1 (0.9)	0.418	0.1 (1.4)	0.6 (1.1)	0.5 (1.3)	0.058	0.258
Cheek									
Right	−43.1 (3.0)	−43.5 (2.9)	−0.4 (2.6)	0.399	−42.2 (2.2)	−42.9 (2.3)	−0.7 (2.2)	0.077	0.464
Left	42.0 (3.2)	42.5 (3.3)	0.5 (2.1)	0.147	41.6 (3.3)	42.3 (3.6)	0.7 (1.9)	0.030	0.694
Total (right + left)^b^	–	–	0.9 (4.1)	0.255	–	–	1.4 (3.1)	0.017	0.348
Upper lip	1.5 (1.7)	1.0 (1.4)	−0.5 (1.5)	0.072	1.0 (1.9)	1.1 (2.0)	0.1 (1.8)	0.673	0.151
Lower lip	0.5 (2.3)	−0.1 (2.2)	−0.6 (1.5)	0.032	0.4 (2.8)	0.6 (2.5)	0.2 (2.2)	0.608	0.950
Angle									
Right	−51.3 (3.0)	−50.5 (3.1)	0.8 (1.4)	0.007∗∗	−49.7 (2.3)	−48.7 (1.8)	1.0 (1.5)	0.001∗∗	0.450
Left	50.5 (4.0)	49.5 (3.6)	−0.9 (1.3)	0.001∗∗	49.4 (3.6)	48.5 (3.5)	−0.9 (1.5)	0.004∗∗	0.902
Total (right + left)^b^	–	–	−1.7 (2.0)	<0.001∗∗∗	–	–	−1.9 (2.1)	<0.001∗∗∗	0.657
Chin	0.7 (2.4)	0.4 (1.7)	−0.3 (2.0)	0.478	0.1 (2.8)	0.1 (1.8)	−0.1 (2.7)	0.853	0.695
Posterior/anterior, mm^c^									
Nose	−14.8 (3.0)	−15.6 (3.0)	−0.8 (1.2)	<0.001∗∗∗	−13.0 (2.1)	−14.1 (2.2)	−1.1 (1.1)	<0.001∗∗∗	0.813
Cheek									
Right	6.1 (2.7)	4.4 (2.9)	−1.7 (1.5)	<0.001∗∗∗	6.8 (3.8)	5.0 (3.0)	−1.7 (2.5)	<0.001∗∗∗	0.989
Left	6.4 (2.9)	4.3 (2.8)	−2.1 (1.7)	0.001∗∗	6.7 (4.3)	5.0 (3.2)	−1.7 (3.2)	0.005∗∗	0.565
Total (right + left)	6.2 (2.8)	4.3 (2.7)	−1.9 (1.3)	<0.001∗∗∗	6.7 (3.9)	5.0 (3.4)	−1.7 (2.6)	0.001∗∗	0.730
Upper lip	−14.4 (4.3)	−13.2 (4.0)	1.2 (2.0)	0.003∗∗	−11.3 (3.1)	−12.4 (2.6)	−1.1 (1.7)	0.001∗∗	<0.001∗∗∗
Lower lip	−16.3 (4.7)	−11.2 (4.2)	5.1 (3.3)	<0.001∗∗∗	−13.1 (3.2)	−9.4 (3.9)	3.7 (3.6)	<0.001∗∗∗	0.135
Angle									
Right	24.4 (4.8)	27.1 (4.2)	2.6 (1.7)	<0.001∗∗∗	25.5 (4.7)	28.4 (4.6)	2.9 (2.7)	<0.001∗∗∗	0.476
Left	25.3 (4.0)	28.1 (3.4)	2.8 (1.8)	<0.001∗∗∗	26.2 (4.3)	29.0 (3.5)	2.8 (2.3)	<0.001∗∗∗	0.033
Total (right + left)	24.9 (4.2)	27.6 (3.7)	2.7 (1.4)	<0.001∗∗∗	25.8 (4.4)	28.7 (3.8)	2.8 (2.3)	<0.001∗∗∗	0.723
Chin	−3.3 (5.2)	1.8 (4.6)	5.2 (4.0)	<0.001∗∗∗	−2.6 (1.8)	2.3 (4.1)	4.9 (3.2)	<0.001∗∗∗	0.610
Superior/inferior, mm^d^									
Nose	−47.3 (3.4)	−47.2 (3.6)	0.1 (1.6)	0.708	−48.7 (3.5)	−48.3 (3.2)	0.4 (1.5)	0.160	0.663
Cheek									
Right	−49.1 (3.5)	−49.8 (3.9)	−0.7 (1.72)	0.028	−51.1 (4.3)	−51.0 (3.8)	0.1 (1.6)	0.721	0.075
Left	−49.2 (3.6)	−49.4 (3.9)	−0.2 (1.9)	0.639	−51.0 (3.9)	−51.0 (3.8)	0.04 (1.8)	0.911	0.675
Total (right + left)	−49.2 (3.5)	−49.6 (3.9)	−0.4 (1.7)	0.191	−51.1 (4.0)	−51.0 (3.7)	0.1 (1.6)	0.804	0.420
Upper lip	−70.5 (4.6)	−71.8 (4.9)	−1.4 (2.2)	0.002∗∗	−71.6 (4.7)	−72.1 (3.5)	−0.4 (2.8)	0.401	0.158
Lower lip	−88.4 (5.8)	−90.8 (6.2)	−2.4 (2.7)	<0.001∗∗∗	−90.5 (9.6)	−89.6 (8.2)	0.8 (4.6)	0.530	0.003∗∗
Angle									
Right	−82.7 (5.2)	−81.4 (5.5)	1.4 (1.4)	<0.001∗∗∗	−84.4 (5.9)	−82.9 (5.3)	1.5 (1.6)	<0.001∗∗∗	0.865
Left	−82.8 (5.0)	−80.9 (5.4)	1.9 (1.4)	<0.001∗∗∗	−84.6 (5.7)	−82.5 (5.6)	2.1 (1.7)	<0.001∗∗∗	0.604
Total (right + left)	−82.7 (5.1)	−81.1 (5.4)	1.6 (1.2)	<0.001∗∗∗	−84.5 (5.7)	−82.7 (5.4)	1.8 (1.2)	<0.001∗∗∗	0.547
Chin	−115.5 (7.2)	−117.8 (7.6)	−2.3 (2.4)	<0.001∗∗∗	−119.6 (8.1)	−116.0 (6.9)	3.7 (3.4)	<0.001∗∗∗	<0.001∗∗∗

Abbreviations: SD: standard deviation; T0: preoperative; T1: at least 1-year postoperative.

∗∗*p* < .01, ∗∗∗*p* < .001; significance between T0 and T1 determined by Wilcoxon signed ranks test or paired t-test, and between groups determined by Mann–Whitney U test or independent t-test.

a Positive value indicates left movement; negative value indicates right movement, with the exception of total cheek and total angles.

b Positive value indicates medial movement; negative value indicates lateral movement.

c Positive value indicates posterior movement; negative value indicates anterior movement.

d Positive value indicates superior movement; negative value indicates inferior movement.

## Discussion

This study is the first to compare regional soft tissue changes in 3D following bimaxillary rotational surgery between clockwise and counter-clockwise mandibular setback for class III overbite and open bite deformities. The face was divided into eight regions affected by bimaxillary OGS according to the osteotomy segments, in order to evaluate outcomes. Analyzing the 3D surface by related data points for all facial regions allowed for a precise evaluation of the region of interest, rather than using the typical anatomical landmarks to measure the linear distance and angle between two 3D objects. Our quantitative assessment showed similar patterns of soft tissue changes in terms of mean distance and direction for both groups except the upper lip, lower lip and chin regions. The surface area of the cheek was increased while the surface areas of the angle and chin were decreased. The null hypothesis was therefore rejected.

Clinically, severe skeletal class III deformity presents with cheek depression and mandibular protrusion and patients are routinely treated with bimaxillary surgery, which includes advancement of the maxilla and setback of the mandible [1]. However, in Asian populations, sagittal movement cannot establish a satisfactory result in most circumstances, due to the protrusive upper lip and inappropriate amount of chin setback through the original occlusal plane [[9], [10], [11]]. Our center uses bimaxillary rotational surgery which enhances aesthetic and functional results by reducing the protrusion of the upper lip and achieving an appropriate amount of setback of the chin. The maxilla is rotated clockwise using Le Fort I posterior impaction; in addition, the mandible is rotated by BSSO setback in either a clockwise or a counter-clockwise direction [6,19].

Prior to surgery, the patient's facial and dental characteristics were evaluated in order to tailor the movement of the mandible during surgery to the needs of each patient. As a result, in order to correct the relatively flat mandibular plane, protrusive mandible and chin of patients presenting with overbite, surgical treatment involved clockwise mandibular setback in order to prevent protrusive chin from insufficient setback of the chin. In contrast, to correct the relatively steep mandibular plane, protrusive mandible of patients with open bite, mandibular setback was required to be counter-clockwise in order to prevent retrusive or double chin from excessive setback of the chin. For both groups, the surgical procedure for the maxilla was comparable; clockwise rotation of the maxilla by posterior impaction for correction of cheek depression and proclined upper incisors due to dental compensation, and improvement of smile arc.

The use of CBCT to acquire images of the maxillomandibular region, which consists of both bony and untextured soft tissue data, usually lacks information for the soft tissues of the nose. In order to overcome the limitation, the combination of both CBCT and 3D stereophotogrammetry was used in this study to produce a 3D dataset consisting of both bony and textured soft tissue information. This dataset has been demonstrated to be accurate and reliable, with an error <1 mm [30], and can be used for the planning and evaluation of facial surgery [35].

Significant advancement of the nose and cheek regions were observed in both groups. Nasal change after maxillary advancement is a major concern to Asian patients who tend to have a wider and flatter nose than Caucasians. Previous studies have mentioned a subjective worsening of the nose appearance including nasal widening and increased nostril show after maxillary advancement in Asians [26]. The current study showed the absence of significant nasal widening, which is consistent with the finding in a study of Chen et al. [36] but incongruent with the other three studies [17,26,37]. This confliction might be associated with different nasal cinch techniques or performance. That is, we had routinely performed alar base cinching during the procedure of intraoral wound closure. The cinching passed through nasalis muscle and anterior nasal spine as reported in the study of Chen et al. [36]. A minor anterior repositioning of the nose region by a mean of approximately 1 mm is corresponding to the finding of forward movement of pronasale in the study of Worasakwutiphong et al. [26]. Asian populations consider a normal cheek contour to be an important characteristic of youth and beauty [19,38]; a flat cheek contour is viewed as unattractive [39,40]. The mean anterior repositioning of the cheek region following surgery was 1.9 mm and 1.7 mm for overbite and open bite groups, respectively. Our results are similar to previous studies on maxillary advancement [24,25] and maxillary rotation by posterior impaction [13,16,19,20]. The anterior repositioning of the cheek region also leads to an increase in area in our study.

Clockwise and counter-clockwise mandibular rotation resulted in mean changes in posterior repositioning of the lower lip (5.1 mm and 3.7 mm, respectively), chin (5.2 mm and 4.9 mm, respectively) and bilateral angles (2.7 mm and 2.8 mm, respectively). These sagittal changes were largest in the chin and lower lip regions, medium in the bilateral angle regions, and smallest in the cheek region. These changes are considered reasonable as they correspond to the amount and direction of the bimaxillary rotational surgery with the maxillary rotation center at the anterior maxilla. Verdenik and Hern [24] also reported that the soft tissue was retracted at the lower lip and chin by a mean of 3 mm, and that it was transversely retracted by a mean of 1.5 mm on the bilateral angles, indicating more changes in the central part of the face than in the lateral parts. In contrast, Lim et al. [41] demonstrated that the sagittal changes exhibited increased gradients from the lower lip to the chin. It should be noted that there was medial and superior repositioning of the bilateral angle regions (mean approximately 1 mm and 2 mm, respectively). This might be explained by (1) bone trimming to remove interference between the proximal and distal segments during BSSO setback for the purpose of satisfactory bone contact between the two segments [42], and (2) angle remodeling after complete detachment of the pterygo-masseteric sling during BSSO setback for the purpose of post-surgical stability [42,43]. Accordingly, both the soft tissue bi-gonial width and angle areas were decreased after surgery. As the areas of the lower third of the face (both angles and chin) decreased after the bimaxillary rotational surgery, the face appeared smaller, which is the current preference of most Asians. The posterior reposition of the bilateral angle regions might result from setback of the mandible.

Three soft tissues differed in repositioning between the two groups. There was posterior and inferior repositioning of the upper lip and inferior repositioning of the lower lip in overbite group compared with anterior repositioning of the upper lip in open bite group, which might be explained by the presence of an abnormal upper and lower lip position from the protruded mandibular incisors before surgery in patients with overbite. The third difference was in the inferior repositioning and superior repositioning of the chin for overbite and open bite groups, respectively. Such difference is likely due to the difference in the surgical rotation of the mandible.

Postoperative changes between T0 and T1 showed decompensation of incisors. The uprighting of the upper incisors was achieved by clockwise rotation of the palatal plane from Le Fort I posterior impaction. The proclination of the lower incisors was achieved by alignment and leveling from post-surgical orthodontic treatment [2,44,45]. The repositioning of the upper and lower lips reported in this study were therefore the long-term results of the combined effects of the rotational surgery and post-surgical orthodontic treatment.

This study has some limitations. First, some patients underwent genioplasty for aesthetic improvements of the chin. However, the frequency was the same for both overbite and open bite deformities. Second, we did not examine whether there was a strong relationship between the regional soft tissue changes and changes in the underlying hard tissues, which will require further study. Third, future studies should also evaluate the changes in the volume for eight regions of the face.

## Conclusions

The manipulation of the maxillomandibular complex in orthognathic surgery has been used in the treatment of class III deformity in order to optimize aesthetic and functional results: mandibular setback clockwise for patients with overbite and counter-clockwise for patients with open bite. In order to provide predictive data for future treatments, it is important to understand the orthognathic outcomes on different facial regions for patients with class III deformity. The results suggest that treatment of class III overbite and open bite deformities with bimaxillary rotational surgery led to comparable regional soft tissue changes, except for the posterior and inferior repositioning of the upper lip and inferior repositioning of the lower lip in overbite group and vertical repositioning of the chin. Anterior repositioning of the nose, increased projection of the cheek, posterior repositioning of the lower lip and chin, and medial and superior repositioning of the bilateral angles following surgery were expected. The surface area of the cheek increased while the surface areas of the bilateral angles and chin decreased. These findings provide a better understanding of the orthognathic outcomes using clockwise or counter-clockwise mandibular setback on different facial regions for patients with class III overbite and open bite deformities.

## Ethical approval

All procedures performed in the study were in accordance with the ethical standards of the institutional research committee and with the 1964 Helsinki declaration and its later amendments or comparable ethical standards.

## Informed consent

Informed consent was obtained from all participants.

## Conflicts of interest

All authors declare that they have no conflicts of interest.
